# Blind Spot for Sedentarism: Redefining the Diseasome of Physical Inactivity in View of Circadian System and the Irisin/BDNF Axis

**DOI:** 10.3389/fneur.2018.00818

**Published:** 2018-10-01

**Authors:** Judit Zsuga, Csaba E. More, Tamas Erdei, Csaba Papp, Szilvia Harsanyi, Rudolf Gesztelyi

**Affiliations:** ^1^Department of Health System Management and Quality Management in Health Care, Faculty of Public Health, University of Debrecen, Debrecen, Hungary; ^2^Department of Psychiatry, Faculty of Medicine, University of Debrecen, Debrecen, Hungary; ^3^Department of Pharmacology and Pharmacotherapy, Faculty of Medicine, University of Debrecen, Debrecen, Hungary

**Keywords:** circadian rhythm, irisin, BDNF, suprachiasmatic nucleus, physical inactivity, entrainment

## Abstract

**Introduction:** The term “diseasome of physical inactivity” was coined by Pedersen to explain clustering of chronic diseases linked to physical inactivity. Accordingly, physical inactivity *per se* contributes to the accumulation of visceral fat, which, generates chronic low-grade systemic inflammation, contributes to emergence of chronic, non-communicable diseases. Diversity of these disorders posits the possible involvement of a supraphysiological system.

**Methods:** Hypothesis driven literature search and deductive reasoning was used to review relevant literature and formulate a novel theory.

**Results:** We have identified the circadian system, omnipresent in virtually every cell, as a possible vehicle for brain muscle crosstalk, explaining some aspects of the diseasome of physical inactivity This system is hierarchically organized, with the suprachiasmatic nucleus (SCN) being the master clock that entrains to the dark/light cycle and synchronizes subsidiary molecular clocks in the periphery. Insufficient photic entrainment also causes chronic disease evolution. The recently identified irisin, was shown to induce brain-derived neurotrophic factor (BDNF) production in several brain areas. BDNF assumes significant role in gating light's influence in the retinohypothalamic synapse, by having a permissive effect on glutamate signal transduction underlying photic entrainment.

**Conclusions:** Here we provide theoretical evidence to support the hypothesis that irisin may facilitate photic entrainment of the SCN, *via* BDNF. By this irisin opens up possible pathways for peripheral non-photic entrainment signals to exert influence on the master clock that is otherwise resistant to these. Furthermore, we suggest that intertwining processes of circadian, redox, inflammatory, and myokine systems lay underneath the diseasome of physical inactivity.

## Introduction

**Figure 1:**
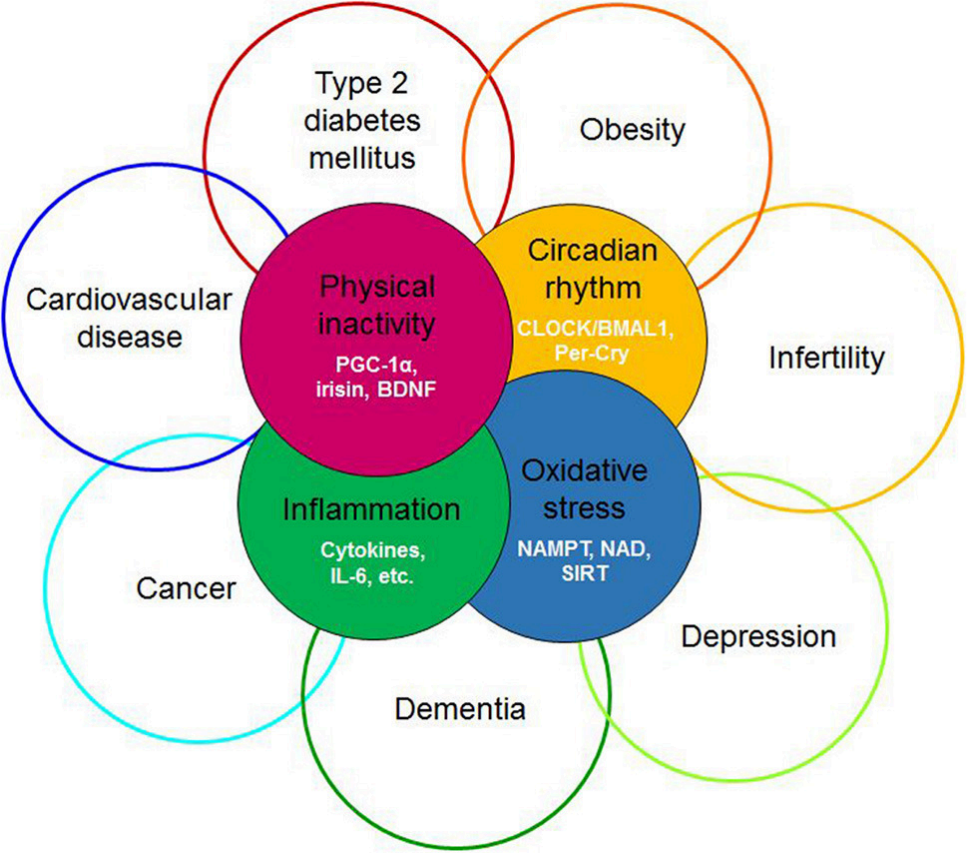
Redefining the diseasome of physician inactivity: evolution of the cluster of diseases defined by the diseasome of physical inactivity due to the derangement of complex interactions between the circadian, the redox, the inflammatory and the muscle derived irisin/BDNF axis. BDNF, brain-derived neurotrophic factor; PGC-1α, peroxisome proliferator-activated receptor γ coactivator 1α; CLOCK, Circadian Locomotor Output Cycles Kaput; BMAL1, brain and muscle ARNT-like protein 1; Per-Cry, period and cryptochrome; IL-6, interleukin 6; NAMPT, nicotinamide phosphoribosyltransferase; NAD, nicotinamide adenine dinucleotide; SIRT1, sirtuin 1.

The term “diseasome of physical inactivity” was coined by Pedersen to explain clustering of chronic diseases linked to physical inactivity (1). This diseasome includes type 2 diabetes, cardiovascular diseases, colon cancer, postmenopausal breast cancer, dementia, and depression (Figure 1). Accordingly, physical inactivity *per se* promotes the accumulation of metabolically active visceral fat, which, by generating low-grade systemic inflammation, contributes to clustering of chronic, non-communicable diseases (1). Cause and effect relationship was demonstrated between physical inactivity and metabolically active visceral fat in healthy young men, who decreased their daily stepping from the generally recommended 10,000 steps/day to 1,500 steps/day, for 2 weeks. This intervention, limiting physical activity, caused an MRI-verified 7% increase in intra-abdominal fat mass without the change in total fat mass. Additionally, parallel decrease of total fat-free mass and body mass index, impaired glucose tolerance, and blunted postprandial lipid metabolism were witnessed (2). These findings indicate that absence of contractile activity of muscle directly contributes to systemic derangements in several organs possibly by releasing soluble mediators. Accordingly, skeletal muscle was coined as an endocrine organ, after myokines, - muscle derived polypeptides with auto-, para- and endocrine function—were discovered and thus offered a mechanism that may explain these elaborate effects (3).

Recently, a novel contraction-regulated myokine, irisin, has been identified, and was shown to have peripheral and central effects (4). Irisin is formed by proteolytic cleavage from the fibronectin type III domain-containing protein 5 (FNDC5). FNDC5 is a transmembrane protein that is expressed under the regulation of the peroxisome proliferator-activated receptor γ coactivator 1α (PGC-1α) (4), an inducible transcriptional co-activator known for its ubiquitous metabolic effect conferred by mitochondrial biogenesis (5). In the periphery, its most significant action is browning of beige adipose tissue by inducing translation of mitochondrial uncoupling protein 1 (UCP1) and consequent non-shivering thermogenesis (4). In mice, the central effect of irisin proved to be induction of brain-derived neurotrophic factor (BDNF) in the hippocampus, an effect seen both in response to elevated peripheral irisin levels and forced expression in cultured primary hippocampal neurons (6, 7). BDNF is known for enhancing the dopamine content in the ventral tegmental area (VTA) and for mediating neuronal plasticity, learning, and memory in limbic structures (8–10) by activating its cognate receptor, the tropomyosin-related kinase B (TrkB) receptor (11). Since both the hippocampus and VTA are involved in motivation and reward processing, our group has proposed a putative role for the irisin/BDNF axis in the evolution of sedentary lifestyle by being on the crossroad of mesocortico-limbic processes and physical activity (12). Nevertheless, the mechanisms underlying physical inactivity and its link to the diverse array of diseases included in the diseasome of physical inactivity remain elusive, giving way to the notion that potential derangement of a supraphysiological system may be involved.

**Figure 2:**
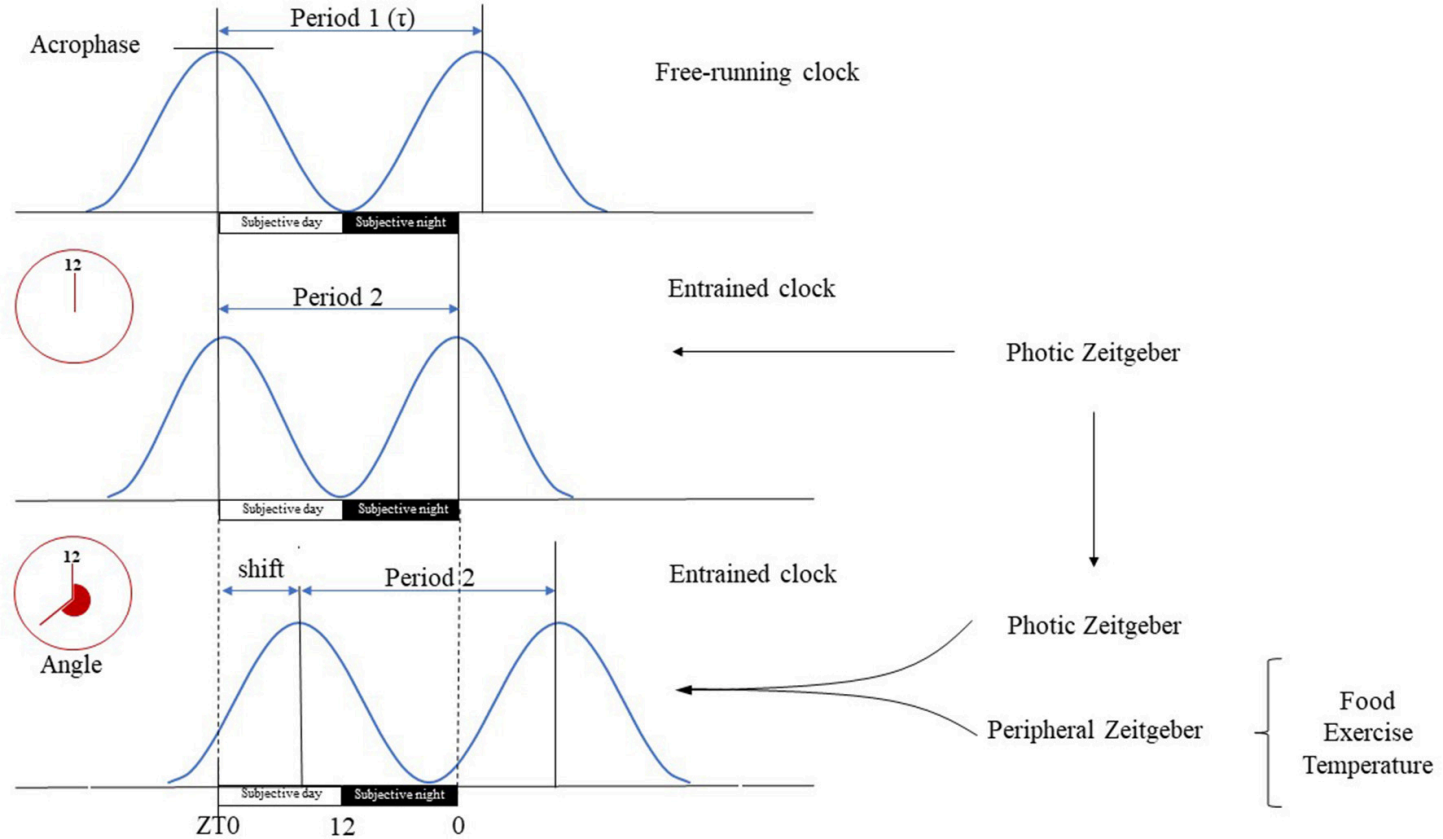
Basic terms of circadian physiology. Dedicated cells of the SCN have self-sustained circadian pacemaker activity that is propagated to other cerebral tissues and peripheral organs. The intrinsic period of these oscillations (τ) is generally longer than the 24-h long geophysical circadian time period. In the absence of Zeitgebers, the master clock is free running, e.g., its period length is unaltered and driven solely by its pacemaker activity. Therefore, the free running clock will be misaligned with the environmental light-day photoperiod, yielding a phase angle of entrainment, e.g., difference between the timing of circadian oscillations and that of the photic input. The magnitude of the phase angle depends on the difference deviation of τ from the 24-h period, the strength of the Zeitgeber and the individual's responsiveness. The SCN is mainly entrained by photic input, e.g., light can align the intrinsic period of oscillations with the environment. Peripheral organs on the other hand have only slave oscillators, e.g., their activity becomes arrhythmic in the absence of entrainment. Their oscillations are entrained by the neural, hormonal etc. outputs of the SCN, and by peripheral, environmental signals specific for the given tissue (e.g., cold, fast, exercise). Tissue specific entrainment may cause a phase shift of peripheral oscillations with respect to the master clock, leading to a discrete displacement of the oscillation along the time axis. Subjective day is the part of the circadian period during which the organism is exposed to illumination (Zeitgeber time 0–12 h), while subjective night is the segment of the circadian period when it is dark (Zeitgeber time 12–24 h).

The circadian system comprises of a network of molecular clocks ubiquitously present in virtually every cell (13) with ~43% of all genes showing circadian rhythmic mRNA expression (14, 15). The omnipresence of the circadian system in light-sensitive organisms posits that adaptation to circadian periodicity (due to the Earth's rotation around its axis) carries a significant evolutionary advantage, possibly by enabling the organism to anticipate regular environmental changes (16, 17). Circadian rhythm has been present throughout phylogenesis with archaebacteria showing primitive circadian oxidoreductive cycles (18, 19). In humans, albeit redox-based clocks are also present (20, 21), circadian rhythm is driven by a cell autonomous core clock machinery comprised of a transcriptional-translational feedback loop (TTFL) (14, 22). On a systems level, molecular clocks show hierarchical organization, with the master clock being located bilaterally in the suprachiasmatic nucleus (SCN) of the ventrolateral hypothalamus (above the optic chiasm, on both sides of the third ventricle) (23). It orchestrates top-down synchronization of tissue specific subsidiary clocks that reside in most organs, including muscle (15, 22, 24). The free-running period of the molecular clocks generally differs from the 24-h day (is usually longer) (25), hence molecular clocks must be entrained by environmental cues (Zeitgebers). Entrainment e.g., adjusting the phase of the molecular clock to be aligned with the timing of an environmental cue, may occur in response to photic (light) and non-photic (food, temperature, exercise, stress, or arousal) environmental stimuli (15). The ability to be entrained is one of the most important properties of the circadian machinery, as this enables adaptation to the environment (22), in fact given its comprehensive regulatory role throughout the body, the light-driven circadian machinery may be considered as the “seventh sense” of perception (complementing the five traditional ones and balance) (Figure 2).

Light/dark, feeding/fasting and resting/exercise are the most significant Zeitgebers reflecting the evolutionary pressure to be adept at locating and attaining food (22). Hence, the circadian machinery is geared to align the diurnal opportunities offered by the environment, with the necessity to allocate mental resources to secure food, at the expense of vigorous exercise, even under circumstances of food scarcity. The master clock is innervated by a specialized light-response pathway, the retinohypothalamic tract, emanating from the retina (26). Light sensitivity of these retino-recipient pacemaker cells shows pronounced circadian fluctuation and are entrained by light (27). In fact, photic entrainment of the SCN, e.g., phase-adjusting ability to light, is the most robust direct entrainment signal of the master clock and downstream molecular clocks located elsewhere in the brain and in peripheral tissues (28). This is reflected by light's ability to reset the intrinsic SCN pacemaker only during subjective night, as light is not able to reset SCN during subjective day, making it possible for the SCN to align circadian behavior with the day and night cycle (29). Photic entrainment is mediated by complex signalization, that includes glutamate, pituitary adenylate cyclase-activating polypeptide (PACAP), and BDNF, with the latter postulated to play a critical role by gating the circadian system to light (18, 30).

The limited capability of the central clock to be entrained is well reflected by plethora of health disturbances caused by the disorganization of the timing system, which leads to circadian misalignment (29). Chronic jet lag, social jet lag, or shift work causes the phase shift of the individual's internal central clock due to objective (e.g., intercontinental travel) or subjective (conflicting social schedule) causes (31–33). In addition, marked lag in internal circadian timing is also defined as delayed sleep-wake phase disorder, by the 5th edition of the Diagnostic and statistical manual of mental disorder In this disorder the biological rhythms governed by the SCN e.g., the sleep-wake cycle, oscillations in core body temperature and endogenous melatonin secretion (18) are considerably delayed with respect to the normal 24 h physical and social environment of a person (33, 34). Preclinical and clinical studies have shown the causative role of circadian misalignment in the evolution of metabolic syndrome (35), obesity and diabetes (36), cardiovascular disease (37), depression (38), prostate and breast cancer (39), and dementia (40, 41). Moreover, a wide array of single nucleotide polymorphisms of clock genes has been identified in metabolic disorders, mood disorders, and cancer (42). Additionally, the pathophysiological implications of distinct (“early bird” and “night owl”) chronotypes, e.g., the preferred phase angles of the central clock with respect to external time, have been extensively studied (43).

Rodent models of chronic jet lag developed by altering the light-dark cycle, corroborated the findings of epidemiological studies, as animals displayed inflammation (44), disrupted microbiome composition (45) accelerated cellular aging (46) mortality (47), diabetes (48), obesity (49), and cancer (50). For example, the causative effect of persistent jet lag in carcinogenesis was elegantly demonstrated by an experiment, in which chronic jet lag was sufficient to induce non-alcoholic fatty liver disease that subsequently progressed to steatohepatitis, fibrosis and hepatocellular carcinoma (51). Moreover, the ability of jet lag to induce obesity by causing dysbiosis was evidenced by fecal transplantation to germ-free mice (45). The overlap between the diseasome of physical inactivity and the scope of diseases emerging in face of circadian misalignment is worthy of notice at this point.

The aim of the current study was to reconceptualize the diseasome of physical inactivity by meticulously reviewing its links with the circadian rhythm. Accordingly, several fields of research are overviewed using hypothesis-driven literature searches and findings are integrated, by means of deductive reasoning to form a novel compelling theory. As the current work is a narrative review it builds mainly on the conceptual and the experimental findings of others, cited throughout the text. Exploratory searches were conducted on PubMed, in English for the following topics: diseasome of physical inactivity, molecular clocks, master clock, peripheral clocks, entrainment, synchronization, oxidoreductive cycle, metabolism, muscle, BDNF, hypothalamus, irisin. Articles of high relevance were identified subjectively and used as starting points for identifying further articles (by reviewing both the articles cited by these and those that have cited them latter) of high impact.

In the forthcoming sections we will compile evidence to support the hypothesis that muscle derived irisin may contribute to circadian alignment, by inducing BDNF expression in the ventrolateral part of the SCN, in a way that it subsequently fine tunes the photic entrainment of the master clock. Furthermore, we offer an elaborate context for interpreting of the diseasome of physical inactivity, complementary to the original concept (1), by suggesting that the intertwining processes of circadian, redox, inflammatory and myokine systems lead to systemic derangements causing the emergence and progression of the wide range disorders and diseases (Figure 1).

## The cellular core clock machinery

The core machinery of the cell-autonomous clocks, residing in virtually every cell, consists of interlacing TTFLs that complete a full cycle over the course of a day. Initially Brain and Muscle ARNT-like Protein 1 (BMAL1) and Circadian Locomotor Output Cycles Kaput (CLOCK), or its homologous protein Neuronal PAS Domain Protein 2 (NPAS2) heterodimerize and bind to the E-box motif in the promoter region of Period (Per1, Per2, Per3) and Cryptochrome (Cry1, Cry2) genes. This drives the transcription of the circadian repressors Per and Cry, that upon their translation in the cytoplasm multimerize and translocate to the nucleus. There, they inhibit BMAL1/CLOCK activity providing a negative feedback mechanism that is completed on a diurnal timescale (32). Ancillary to this core loop, BMAL1 transcription may be inhibited by the nuclear orphan receptor REV-ERBα elements, proposed to be an integrator of metabolism and circadian rhythm (42, 52, 53). Periodic expression of core clock genes in the SCN is mediated by calcium/cAMP response binding protein (CREB) regulatory elements that are activated by membrane depolarization, increased intracellular calcium level and cAMP (53). Phosphorylated (activated) CREB in turn binds to calcium/cAMP regulatory elements (CREs) on the promoter sequence of and Per1 and Per2 (53).

Parallel to driving circadian oscillations, transcription factors of the TTFL regulate the rhythmic expression of numerous clock-controlled genes (CCGs) involved in diverse physiological processes, causing temporal regulation of metabolic and coupled oxidoreductive processes (24). Moreover, molecular clocks serve to optimize the tissue-specific timing of cellular processes, offering a mechanism to temporally separate biochemical processes. These, which if superimposed would be incompatible and lead to deleterious consequences, e.g., increased mutation rate (16). In line with this, is the observation that for example, transcripts of key cellular/metabolic genes are highly enriched in the circadian transcriptome of skeletal muscle (54, 55). The muscle plays a central role in whole-body metabolism, hence its metabolic processes are all aligned with and regulated by the circadian cycle (24). Accordingly, the muscle molecular clock regulates the activation and repression of the rate-limiting metabolic genes temporally. Furthermore, the muscle clock synchronizes with the central clock, but also may be entrained by feeding and physical activity. This allows skeletal muscle to anticipate regular environmental circadian oscillations as well as to adapt to individual metabolic challenges (29). This is of utmost significance as the skeletal muscle is responsible for cc. 80% of insulin-stimulated post-prandial glucose uptake, and it is a reservoir of amino acid used for protein synthesis and gluconeogenesis (54).

As the alternating glycolytic-oxidative energetic cycles show transcription dependent circadian periodicity, the need to identify cofactor signaling molecules that link the metabolic and circadian systems has emerged. For example, both nicotinamide phosphoribosyltransferase (NAMPT), the rate-limiting enzyme in NAD^+^ biosynthesis and consequently the metabolic cofactor nicotinamide adenine dinucleotide (NAD^+^) show circadian rhythm (56, 57). NAMPT is upregulated by CLOCK, leading to diurnal fluctuations in NAD^+^ levels. NAD^+^ in turn binds and activates the NAD^+^ dependent histone deacetylase sirtuin 1 (SIRT1) (58), rendering its activity circadian as well (59). SIRT1 is regulated by the ratio of NAD^+^ to NADH making it a bioenergetic sensor (5). It was shown to deacetylate genes involved in the positive (60) and the negative (61) limb of the circadian core clock machinery, and the transcriptional regulatory proteins of metabolic and inflammatory pathways i.e. PGC-1α and Nuclear Factor κB (NFκB) (59). Thus NAMPT-regulated NAD^+^ biosynthesis *via* its downstream mediator SIRT1 may fine-tune and align diurnal cycles of energy storage and utilization with the rest-activity cycle.

Nevertheless, reports have indicated that deacetylation by SIRT1 may impose opposing effects on the molecular clock. On one hand, Ramsey et al. (57) have shown that deacetylation of BMAL1 suppresses the CLOCK/BMAL1 complex (57) in the hepatocytes, thus it functions as a negative regulator. Conversely, inhibition of NAMPT liberates the CLOCK/BMAL1 complex from SIRT1 suppression (14, 59). On the other hand, SIRT1 mediated deacetylation of Per2 leads to Per2 ubiquitination and degradation, rendering SIRT1 a positive regulator (61). Furthermore, SIRT1 and PGC-1α bind cooperatively to BMAL1 promoter, and increase the amplitude of BMAL1/CLOCK gene expression in the SCN of mice (59).

As indicated, PGC-1α, is an important component of the mammalian clock. It is able to induce BMAL1 and CLOCK gene expression and translation (52) by modifying the chromatin microenvironment from repressive to active state. Expression of PGC-1α is rhythmic (62, 63), furthermore its deacylation by SIRT1 (52, 64) renders its activity to follow a circadian rhythmic pattern as well. Since PGC-1α expression is further induced by signals that convey metabolic need e.g., cold, fast, and exercise (65–67), the critical role of PGC-1α in linking environmental demands to metabolic needs and synchronizing these with the circadian rhythm seems feasible. The circadian amplifying loop, comprised of NAMPT, SIRT1, and PGC-1α influences the intrinsic period of the master clock. This finding was evidenced by genetic models (the period length shortens and elongates in transgenic mice with increased SIRT1 and in brain-specific SIRT1 knockout mice, respectively) (59). Thus it follows that this loop assumes a critical role in the SCN central pacemaker (59). Summarizing it seems feasible to suggest that PGC-1α may serve as a significant factor mediating the entrainment of molecular clocks by linking the effects of environmental signals that offer evolutionary advantage (e.g., caloric restriction, exercise, cognitive demand) to the core clock machinery.

## Hierarchical organization of the circadian system, entrainment and synchronization

The most fundamental environmental signal, profoundly determining physiology and behavior of diurnal mammals, is derived from the daily fluctuation of light and dark periods. To accommodate, the mammalian timing system is hierarchically organized in a way that the master clock residing in the SCN, is synchronized to geophysical time. This is done mainly through photic signals delivered *via* the retinohypothalamic tract (18). The retinohypothalamic tract is a monosynaptic tract that emanates from intrinsically photosensitive retinal ganglion cells, which contain the blue light sensitive photopigment, melanopsin (68). It ends on a subset of neurons in the ventrolateral part of the SCN that express vasoactive intestinal peptide (VIP) (18). This part of the SCN, the core, has an intrinsic pacemaker activity that drives the synchronization of the oscillations in the rest of the SCN, through synaptic cell to cell coupling and paracrine signalization (18, 53, 69). In fact, cell to cell coupling enables SCN cells to remain coordinated even in the absence of external time cues (69) by synchronizing and enhancing the precision of component cellular oscillation and offering robustness to genetic perturbations (53). In addition, this internal coupling leads to the coherent output signal of the SCN even if it is free-running (53), e.g., driven solely by its pacemaker activity. Nevertheless, in most species, this free-running intrinsic circadian period is longer than the 24 h long environmental photoperiod (22), hence continuous adjustment (entrainment) is mandatory. Inappropriate entrainment of the master clock leads to the evolution of varying diseases and disorders (see before). The SCN is most readily entrained by light, however resent reports posit that exercise and restricted feeding may also be of some, albeit considerably less influence (15, 29, 33, 70). Entrainment of the SCN to light, by its retinal innervation directly modifies the timing, amplitude, period, and phase of the electrical activity of the SCN pacemaker. This light derived information is directly translated into change of the rhythmic expression pattern of the TTFL of the core clock machinery (17, 22). This in turn enhances daily oscillations of CCG expression (28) leading to optimized locomotor activity, metabolism and energy balance (16, 22). The SCN transmits the environmental rhythm to other areas of the brain and the peripheral organs *via* its neuronal connections, by altering sympathetic outflow (16), endocrine signals (e.g., melatonin) (31, 43), body temperature rhythm, and other indirect cues (18, 28). Hence, the SCN synchronizes peripheral oscillators to enable the organism to anticipate daytime and organize physiology and behavior proactively and to ascertain circadian regularity (18).

Intrinsic molecular clocks of the peripheral organs are synchronized by the SCN and are also entrained by Zeitgebers relevant for their tissue-specific physiology (22). Thus, peripheral clocks show distinct pattern of phase shifts with regards to that of the master clock, as a net of indirect synchronization to light by the SCN and entrainment by peripheral signals (29). Peripheral circadian clocks directly respond to restricted feeding schedules and exercise, enabling entrainment to optimize temporal fine-tuning of tissue specific metabolic processes. Nevertheless, the intrinsic rhythm of most subsidiary oscillators, the retina and the olfactory bulb being exceptions (71, 72), gradually damp out in the absence of the SCN (53, 73) rhythm. Thus, optimal entrainment of peripheral clocks ensues when molecular clocks in brain areas outside the SCN and peripheral clocks in the liver, muscle, pancreas, lung, kidney, bone etc. align their characteristic internal phase with respect to the core clock and each other in a way that their tissue-specific function is temporally optimized and serves maximal adaptation to the 24-h environment (22).

For example, the muscle clock is readily entrained by motoneuron-dependent contractile activity and feeding/fasting (54, 63, 74). Thus, CCG regulate the time-of-day shifts in carbohydrate and lipid metabolism, by means of temporal regulation of genes, involved in rate-limiting steps for catabolic and anabolic processes in skeletal muscle. Accordingly, a shift from lipid to carbohydrate catabolism is induced during the early active period, due to peak expression of genes that regulate glycolysis and glycolytic flux into the Kreb's cycle. This is subsequently followed by a peak in the expression of genes involved in carbohydrate storage and lipogenesis at the end of the active phase, when storage of excess energy prevails in the postabsorptive stage. Following, genes involved in fatty-acid catabolism are activated during the mid-inactive period, providing lipids as major energy source for the skeletal muscle during the overnight fast (24). Genetic models also reflect the importance of maintaining circadian rhythm in skeletal muscle for metabolic homeostasis of muscle. Findings derived from two models that have decreased muscle specific transcript of BMAL1, the only non-redundant core clock gene (74), indicated that BMAL1 regulates both glucose uptake and metabolism in muscle. This supports the transition from the rest/fast phase to the active/feeding phase in a coordinated manner, so glucose can serve as the principal fuel in muscle (74, 75). Furthermore, the muscle molecular clock modulates the transcription of over 2,300 genes (54, 76), including PGC-1α and FNDC5 (13), rendering their expression also circadian.

Summarizing the SCN may be conceptualized as the key structure underlying the “seventh sense” of organisms (complementing the visual, auditive, tactile, olfactory, taste, and vestibular senses), as it offers an additional sensory modality. This enables adaptation to the environment and regulation of fundamental cellular processes in a tissue specific manner (e.g., the redox state, inflammatory state, metabolic state). Furthermore, current knowledge posits that the SCN is most readily entrained by photic input received at night and shows limited response to non-photic entrainment signals.

## Molecular mechanisms of photic entrainment

Given that the SCN may be conceptualized as the conductor of an orchestra aligning every peripheral clock, it's appropriate entrainment by photic stimuli is fundamental, to permit synchrony between physiology, behavior, and the surrounding environment. Photic entrainment is confined to the VIP containing neuronal cells in the SCN that have the molecular machinery to convert light derived information to chemical signal (18, 30). Light transmitted *via* the retinohypothalamic tract leads to the release of glutamate and PACAP, the primary neurotransmitters underlying photic regulation of the SCN (18, 77, 78). These upon activating their specific receptors, the NMDA (N-methyl-D-aspartic acid) and PAC1 (pituitary adenylate cyclase-activating polypeptide type 1) receptor (79), respectively, lead to the increase of postsynaptic cAMP and Ca levels, that will manipulate the peak/phase expression of genes in the core molecular clock and downstream clock-controlled genes (54, 80, 81). Synaptic transmission in the retinohypothalamic tract recipient area of the SCN is further modulated by the BDNF-TrkB system (82).

NMDA receptor activation leads to phosphorylation of extracellular signal-related kinase (ERK), a key event of photic entrainment during subjective night (82). Increase of ERK phosphorylation by ras leads to its transient activation and translocation into the nuclear compartment on a timescale that is in line with that of photic entrainment (82, 83). Several upstream signalization pathways in the SCN converge on ERK. NMDA receptors in the SCNs are physically associated with ras, thus NMDA receptor activation leads to ERK phosphorylation (84–86). BDNF-TrkB binding and PACAP also activate ras (82). Furthermore, ligand gated opening of the NMDA receptor leads to increased intracellular Ca level, which also enhances the activation of the ras-ERK pathway (82). Downstream mediators of ERK include BMAL1, that may be directly phosphorylated by ERK (82, 87), as well as CREB, which upon its phosphorylation activates CRE-mediated transcription of the core clock proteins Per1 and Per2 and the light-inducible immediate early genes c-fos, JunB etc. (genes that also have CRE in their promoter regions) (53, 82). *In vivo* studies have underscored the significance of ERK and CREB in the phase shifting effect of light, as disruption of either has led to attenuation of photic entrainment (83, 88–90). Accordingly, CREB is phosphorylated within minutes of a light pulse, leading to subsequent induction of Per1 within 5–15 min if administered during subjective night (53). Promoters of c-fos, JunB, and other immediate early genes also contain CREs and are also induced by light, albeit more slowly (91).

## BDNF, the gatekeeper of photic input in SCN

BDNF is a potent neurotrophin that is synthetized in a pre-pro form and released into the circulation either as pro-BDNF (which is then converted to BDNF by plasma tissue plasminogen activator) or as mature BDNF (the active form, referred to as BDNF further on) (11). It is able to cross the blood-brain barrier and exerts central and peripheral effects (1, 11), in general by activating genes of regulatory proteins involved in cytoskeletal and synaptic plasticity, cell survival, energy homeostasis, and mitochondrial biogenesis (11, 65). Accordingly BDNF assumes a significant function in maturation, and plasticity of the brain (92), by strengthening synaptic connections (93). It facilitates synaptic transmission by modifying the activation kinetics of NMDA receptors and/or by increasing the number of docked vesicles in the synapse (65), evoking long-term potentiation and adaptive modification of neuronal circuits (93) inherent behavioral adaptation (94). BDNF was shown to increase the number of dendritic spines and the complexity of dendrites in CA1 hippocampal neurons and the dentate gyrus, respectively (65). Increase of the number of mitochondria is mandatory to support the formation of new, functioning synapses (5). In light of this, the fact that BDNF upregulates PGC-1α, a regulator of mitochondrial biogenesis, is not surprising. Furthermore, as PGC-1α also enhances BDNF expression, a bidirectional positive feedback mechanism is suggested (6). Conversely, BDNF has emerged as a possible mediator of cognition-enhancing effects of exercise and intermittent fasting (95), a finding underscored by simultaneous increase of BDNF expression in multiple brain regions and improved cognition in response to voluntary aerob exercise and/or intermittent fasting (96–98). The finding that β-hydroxybutyrate, a ketone produced during fasting and exercise, induces BDNF expression in hippocampal cell culture (5, 99) also corroborates this link. Conversely, the ability of irisin to upregulate cerebral BDNF expression in an exercise-responsive manner (6) also supports this notion. BDNF expression was evidenced in varying areas of the brain including the prefrontal cortex, the hippocampus, the amygdala, the VTA (6, 95), structures known for mediating reward-related reinforcement learning consequent change of behavior (100, 101) and in the hypothalamus (95), hosting the master clock in the SCN. The similar distribution pattern of BDNF and FNDC5 is worthy of notice.

Expression and release of BDNF is stimulated by excitatory synaptic activity (11, 102), by neuropeptides and by irisin (11, 12). For example, release of glutamate, the ubiquitous excitatory neurotransmitter, by binding to NMDA receptors leads to the increase of intracellular calcium level, which activates Ca^2+^/calmodulin-dependent protein kinase (CaMK), protein kinase C and ERK (82). These in turn increase cAMP-responsive element-binding protein (CREB) and NFκB. These induce BDNF gene transcription and subsequent transport of BDNF into vesicles (11). BDNF acts by binding to its cognate receptor TrkB, a tyrosine kinase receptor (11).

BDNF *via* activation of TrkB receptor assumes a significant functional role in photic regulation of the SCN (30). Retinohypothalamic fibers terminating in the SCN express TrkB receptors pre- and postsynaptically, in the vicinity of BDNF-expressing SCN cells, thus the spatial relationship is appropriate for modulating this synaptic interaction (30, 103). Accordingly, BDNF was shown to enhance glutamate and PACAP release (30, 82, 103). It also augments postsynaptic response to glutamate by NMDA receptor phosphorylation *via* increasing the opening probability of the channel in mice and rat (85, 103, 104) and/or by inducing rapid cycling of the NMDA receptor, hence increasing the number of NMDA receptors on the cell surface (103). [Conversely, BDNF's ability to increase presynaptic liberation of glutamate via TrkB receptor activation in the hippocampus and visual cortex was also reported previously (18, 105, 106)]. BDNF expression within the SCN is rhythmic, showing basal and elevated BDNF levels during subjective day and night, respectively (107). On one hand, BDNF signaling during subjective day is insufficient to permit excitatory neurotransmitter release, inhibiting the transmission of the light signal through the retinohypothalamic tract-SCN synapse (30). On the other hand, elevated BDNF levels during subjective night *via* activating the TrkB receptor is sufficient to induce functional and structural changes that lead to potentiation of the light-induced retinohypothalamic activation in the SCN (30, 108). These findings are in line with the observations that photic input is unable to entrain the master clock during subjective day, rendering the SCN insensitive to perturbation by light, while the SCN responds to light by prominent phase shifts during subjective night (30). Further corroborating evidence is that administration of BDNF into the SCN of rats alters the circadian light reaction by enabling phase shifts in response to light during subjective day (108, 109). Conversely, photic entrainment is abated in BDNF-deficient knockout mice and in rats infused the tyrosine kinase inhibitor K252a into their SCN (30, 108). Moreover, the findings of Michels and colleagues have shown that BDNF/TrkB signaling is sufficient *per se* to induce phase shifts in the rhythm of neural activity recorded in isolated SCN of mice (103). This suggests the possibility that BDNF may assume a more direct role in photic entrainment by inducing rather than modulating phase shifts.

**Figure 3:**
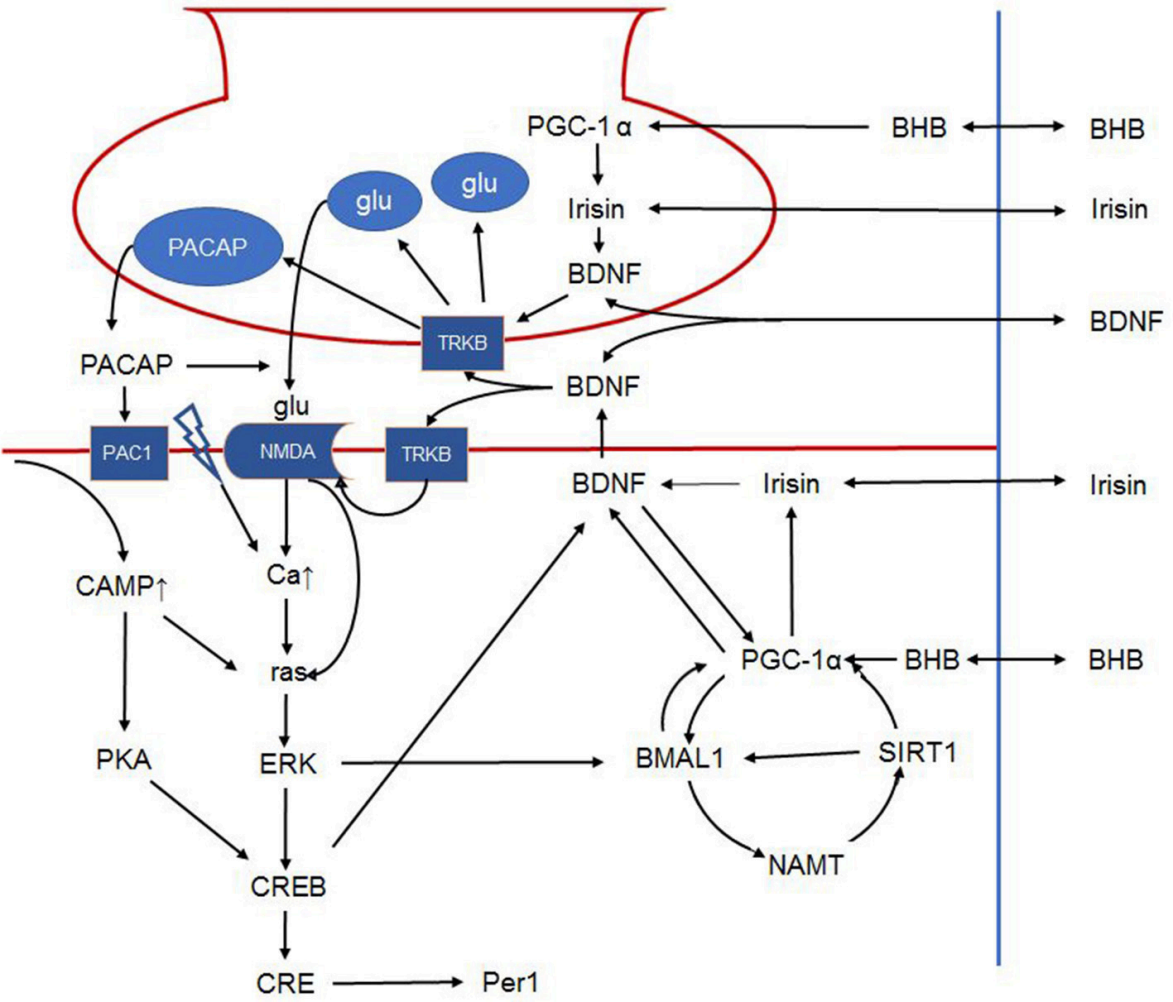
Schematic outline of the intracellular signalization in the retinohypothalamic tract. Presynaptically, BDNF can increase glutamate and PACAP content of synaptic vesicles and facilitates their release. Postsynaptically, BDNF phosphorylates the NMDA receptor, increasing its number and the opening probability of the channel. BDNF furthermore enhances PACAP release. PAC1 receptor activation activates CREB via the cAMP-protein kinase A pathway. NMDA receptor activation leads to ras activation directly and indirectly by increasing intracellular calcium. Ras in turn activates ERK that on one hand activates CREB, and on the other phosphorylates BMAL1. In addition, BMAL1 is regulated by the NAMPT-SIRT1, PGC-1α cycle. Peripheral signals may further influence these complex interactions. For example, BHB induces PGC-1α that in turn induces irisin expression. Irisin's influence on BDNF expression and release is hypothetical in the SCN, however it was described in other brain areas. Hence, in the present article, we propose that peripheral signals (BHB, irisin etc.) liberated in response to non-photic environmental signals may modulate the permissive effect of BDNF. The link between irisin and BDNF in the SCN is hypothetical. BDNF: brain-derived neurotrophic factor; PACAP: pituitary adenylate cyclase-activating polypeptide; NMDA, N-methyl-D-aspartic acid; PAC1, pituitary adenylate cyclase-activating polypeptide type 1 receptor; CREB, cAMP response element-binding protein; cAMP, cyclic adenosine monophosphate; ERK, extracellular signal–related kinase; BMAL1, brain and muscle ARNT-like protein 1; NAMPT, nicotinamide phosphoribosyltransferase; SIRT1, sirtuin 1; PGC-1α, peroxisome proliferator-activated receptor γ coactivator 1α; BHB, β-hydroxybutyrate; SCN, suprachiasmatic nucleus.

Summarizing, modulation of the synaptic transmission between the retinohypothalamic tract and the ventrolateral core of the SCN by BDFN-TrkB signalization is 2-fold, as it involves enhanced presynaptic release of glutamate and postsynaptic increase of NMDA mediated receptor response to glutamate. It is suggested that availability of BNDF is mandatory for glutamate to induce the signal-transduction cascade that mediates the light induced shift of the circadian system. This places BDNF in the focus of photic entrainment of the master clock (30) by attributing a critical role to BDNF in terms of gating the circadian system to light (Figure 3) (103).

## FNDC5/irisin, a putative link between photic and non-photic entrainment signals

Irisin, a putative contraction-regulated myokine is a highly conserved 12 kDa polypeptide identified in mammals including humans (4). Its amino acid sequence shows 100% homology among most mammals, suggesting an evolutionary preserved function (110). The precursor of irisin is FNDC5, a glycosylated type I membrane protein regulated by PGC-1α. PGC-1α is the key regulator of mitochondrial biogenesis that activates downstream transcriptional factors, leading to upregulation of oxidative phosphorylation, mitochondrial DNA replication/transcription, and mitochondrial protein import (52, 111). It enhances the expression of antioxidant enzymes, DNA repair enzymes, anti-apoptotic proteins, protein chaperones and Ca^2+^-handling proteins *via* NFκB and CREB (5). Additionally, it seems that PGC-1α plays a pivotal role in preserving and reorganizing neuronal circuits, a process underlying neuronal plasticity and learning needed for resilience and adaption to environmental challenges. It was shown that synaptogenesis in embryonal hippocampal neurons is dependent on BDNF induced enhancement of PGC-1α expression and subsequent mitochondrial biogenesis at the site of dendritic spine and synapse formation (65).

Expression of FNDC5 and its upstream regulator PGC-1α is induced by endurance exercise (112, 113) and cold (114). Albeit its expression is most significant in skeletal muscle, it has been detected in adipose tissue, tongue, rectum, kidney, and brain (110, 115). Irisin is formed from its precursor by proteolytic cleavage, an effect triggered by exercise and is released into the circulation (4, 6, 7), where it readily crosses the blood-brain barrier (116).

The most renowned effect of irisin in the periphery is upregulation of the expression of thermogenic genes such as mitochondrial uncoupling protein UCP-1, an effect possibly mediated by p38 mitogen-activated protein kinase (p38 MAPK) and ERK pathways (117). This leads to a metabolic switch that results in browning of white adipocyte tissue, parallel to the activation of oxygen consumption and thermogenesis of fat cells (4, 112).

Additional to its profound effect in the periphery, irisin's central effects have also been characterized (111). FNDC5 mRNA was isolated in several structures, such as the midbrain, the hippocampus (116), and the cerebellum (118) of rodents. Irisin is present in the cerebrospinal fluid of humans (119), and in the human hypothalamus (119, 120), showing concentrated expression in the neuropeptide Y containing cells of the paraventricular nucleus (119). Corroborating evidence has indicated that FNDC5/irisin may mediate the beneficial effect of endurance exercise (95), as it leads to elevated neuronal FNDC5 expression in the hippocampus of mice (6, 116), paralleled by increase of PGC-1α level in several corresponding areas, the frontal lobe, hippocampus and midbrain (121). Further evidence also underscores the possibility that PGC1α is an upstream regulator of FNDC5 expression in the brain, similar to muscle, as the level of FNDC5 expression was shown to be reduced in PGC-1α ^−/−^ mice (6).

**Figure 4:**
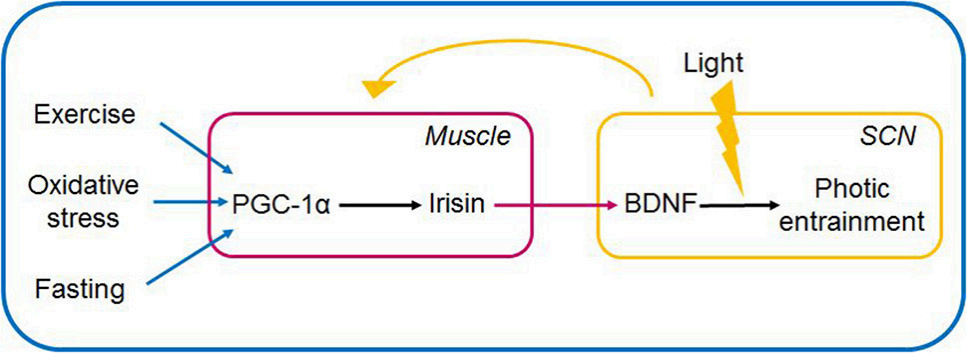
Muscle-brain crosstalk: irisin (via PGC1-α) serves as link between peripheral non-photic entrainment signals (fast, cold, exercise) and light-induced signalization in the SCN. Irisin is induced and released in the periphery, and upon its entering the hypothalamus is hypothesized to induce BDNF expression and release. BDNF by its permissive effect is able to gate the signalization in the SCN if light signal provided. The SCN in turn entrains peripheral organs via neurohumoral, hormonal, temperature, and other indirect signals. The link between irisin and BDNF in the SCN is hypothetical. BDNF, brain-derived neurotrophic factor; PGC-1α, peroxisome proliferator-activated receptor γ coactivator 1α; SCN, suprachiasmatic nucleus.

Irisin in the brain is able to induce BDNF expression at varying sites e.g., in the hippocampus, and the VTA. Elevation of the circulating level of irisin was shown to induce BDNF expression in the hippocampus, as was forced hippocampal expression of FNDC5. Moreover, decrease of cortical BDNF expression was evidenced, following mRNA expression-mediated knockdown of FNDC5 expression (6). Another stream of experiments further articulated the possibility of irisin serving as a cross-organ messenger linking skeletal muscle and brain to enable the organism to react adaptively to its environment. Accordingly, irisin's direct locomotor activity was proposed, when central administration of irisin into the third ventricle of rats, lead to an increase of physical activity (characterized by increase of total travel distance, ambulatory count and time, vertical counts in treated rats vs. controls receiving IgG Fc peptide) (120). Summarizing the above findings, we put forward the speculative notion that the exercise-related irisin mediates brain-muscle crosstalk by possibly linking sedentary lifestyle and circadian rhythms (Figures 3, 4).

This hypothesis is underscored by recent findings that seem to contradict earlier notions regarding the inability of the SCN to be entrained by non-photic Zeitgebers (15, 29). Nevertheless, in order to generate a coherent rhythm, the SCN must integrate environmental Zeitgebers (mainly light) as well as information from the peripheral tissues (18). The presence of sufficient signalization for entraining the central clock by peripheral signals, e.g., exercise and feeding/fasting, is indicated by the ability of exercise and feeding to induce shifts in the SCN under constant darkness (15, 122–124). Albeit light is the main entraining stimuli for the SCN, restricted feeding schedules, hypocaloric feeding (15), and scheduled exercise (15, 125) can also entrain the SCN to a limited extent, under dark-light conditions. Human studies have shown that the combination of light and exercise is needed for entraining the SCN. This is indicated by finding that the sleep-wake cycle may be entrained by the sleep schedule alone, while phase advancement of the circadian rhythm of melatonin was dependent on both the sleep-wake cycle and exercise (126, 127). Conversely, seeking morning bright light, and early morning exercise was proposed to induce phase advances of the master clock in teenagers suffering from delayed sleep-wake phase disorder (33, 128, 129). Alternately, daytime restricted feeding combined with caloric restriction was able to entrain the master clock implicating that metabolic cues liberated in response to caloric restriction may contribute to entrainment. Possible pathways involved in entrainment may be the activation PGC-1α by increase of free fatty acid synthesis and β-hydroxybutyrate, or alternatively the change of the NAD^+^/NADH ratio and associated SIRT1 function. Taking these observations one step further PGC-1α, directly by local expression or indirectly *via* induction of irisin synthesis and release may be hypothesized to increase BDNF levels to the extent that BDNF may facilitate glutamate transmission in the SCN allowing subsequent entrainment.

## Conclusion

Summarizing, we propose that the diseasome of physical inactivity evolves due to the derangement of complex interactions between the circadian system, the redox homeostasis, inflammation and the PGC-1α/irisin/BDNF axis. Furthermore, based on previous findings, that sufficiently high BDNF levels are mandatory for photic entrainment, rendering BDNF as the gatekeeper regulating light's ability to shift the phase of the master clock, we propose that irisin, either by crossing the blood-brain-barrier, or by its direct expression in the hypothalamus is able to increase BDNF levels in the retinohypothalamic synapse, and hence may modulate BDNF's effect on photic entrainment. This is supported by the fact that peripheral effects *per se* are unable to shift the master clock, however simultaneous presence of light and peripheral factors (exercise, and fasting, both known to induce PGC-1α and consequently irisin expression) may do so. Nevertheless, the need for further investigation into the possible role of the irisin/BDNF axis in linking sedentary lifestyle and circadian misalignment are need. In the current work we suggest, that muscle-brain crosstalk enables non-photic entrainment signals to fine-tune the master clock, by inducing the release of the irisin into the systemic circulation. We hypothesize that irisin after crossing the blood-brain barrier in turn increases the expression and release of BDNF in the pre- and postsynaptic terminals of the retinohypothalamic tract, facilitating photic entrainment in the SCN in response to the peripheral signals of fasting, and exercise (Figure 4).

## Author contributions

JZ conceptualized the message of this work and wrote the first draft of the manuscript, CM and CP contributed to the literature processing, TE and SH made the figures, RG finalized the manuscript.

### Conflict of interest statement

The authors declare that the research was conducted in the absence of any commercial or financial relationships that could be construed as a potential conflict of interest.
